# Dietary Fibre May Mitigate Sarcopenia Risk: Findings from the NU-AGE Cohort of Older European Adults

**DOI:** 10.3390/nu12041075

**Published:** 2020-04-13

**Authors:** Diego Montiel-Rojas, Andreas Nilsson, Aurelia Santoro, Claudio Franceschi, Alberto Bazzocchi, Giuseppe Battista, Lisette C. P. G. M. de Groot, Edith J. M. Feskens, Agnes Berendsen, Barbara Pietruszka, Olga Januszko, Susan Fairweather-Tait, Amy Jennings, Claudio Nicoletti, Fawzi Kadi

**Affiliations:** 1 School of Health Sciences, Örebro University, 702 81 Örebro, Sweden; diego.montiel@oru.se (D.M.-R.); fawzi.kadi@oru.se (F.K.); 2 Department of Experimental, Diagnostic and Specialty Medicine, Alma Mater Studiorum, University of Bologna, 40126 Bologna, Italy; aurelia.santoro@unibo.it (A.S.); claudio.franceschi@unibo.it (C.F.); g.battista@unibo.it (G.B.); 3 Interdepartmental Centre “L. Galvani”, Alma Mater Studiorum, University of Bologna, 40126 Bologna, Italy; 4 Department of Applied Mathematics, Institute of Information Technology, Mathematics and Mechanics (ITMM), Lobachevsky State University of Nizhny Novgorod-National Research University (UNN), 603950 Nizhny Novgorod, Russia; 5 Diagnostic and Interventional Radiology, IRCCS Istituto Ortopedico Rizzoli, 40136 Bologna, Italy; alberto.bazzocchi@ior.it; 6 Department of Human Nutrition and Health, Wageningen University, 6708WE Wageningen, The Netherlands; lisette.degroot@wur.nl (L.C.P.G.M.d.G.); edith.feskens@wur.nl (E.J.M.F.); agnes.berendsen@wur.nl (A.B.); 7 Department of Human Nutrition, Warsaw University of Life Sciences-SGGW, 02-776 Warsaw, Poland; barbara_pietruszka@sggw.pl (B.P.); olga_januszko@sggw.pl (O.J.); 8 Norwich Medical School, University of East Anglia, Norwich NR4 7TJ, UK; S.Fairweather-Tait@uea.ac.uk (S.F.-T.); Amy.Jennings@uea.ac.uk (A.J.); 9 Gut Health Institute Strategic Programme, Quadram Institute Bioscience, Norwich NR4 7UQ, UK; claudio.nicoletti@unifi.it; 10 Department of Experimental and Clinical Medicine, Section of Anatomy, University of Florence, 50134 Florence, Italy

**Keywords:** muscle mass, exercise, metabolic syndrome, systemic inflammation, C-reactive protein, protein intake

## Abstract

Sarcopenia is characterised by a progressive loss of skeletal muscle mass and physical function as well as related metabolic disturbances. While fibre-rich diets can influence metabolic health outcomes, the impact on skeletal muscle mass and function is yet to be determined, and the moderating effects by physical activity (PA) need to be considered. The aim of the present study was to examine links between fibre intake, skeletal muscle mass and physical function in a cohort of older adults from the NU-AGE study. In 981 older adults (71 ± 4 years, 58% female), physical function was assessed using the short-physical performance battery test and handgrip strength. Skeletal muscle mass index (SMI) was derived using dual-energy X-ray absorptiometry (DXA). Dietary fibre intake (FI) was assessed by 7-day food record and PA was objectively determined by accelerometery. General linear models accounting for covariates including PA level, protein intake and metabolic syndrome (MetS) were used. Women above the median FI had significantly higher SMI compared to those below, which remained in fully adjusted models (24.7 ± 0.2% vs. 24.2 ± 0.1%, *p* = 0.011, η^2^p = 0.012). In men, the same association was only evident in those without MetS (above median FI: 32.4 ± 0.3% vs. below median FI: 31.3 ± 0.3%, *p* = 0.005, η^2^p = 0.035). There was no significant impact of FI on physical function outcomes. The findings from this study suggest a beneficial impact of FI on skeletal muscle mass in older adults. Importantly, this impact is independent of adherence to guidelines for protein intake and PA, which further strengthens the potential role of dietary fibre in preventing sarcopenia. Further experimental work is warranted in order to elucidate the mechanisms underpinning the action of dietary fibre on the regulation of muscle mass.

## 1. Introduction

Sarcopenia is characterised by a progressive loss of skeletal muscle mass accompanied by the deterioration of physical function, culminating in the loss of independence and increased morbidity risk [1,2,3]. The occurrence of metabolic abnormalities, characterised by elevated fasting glucose and triglyceride levels, reduced HDL-cholesterol and increased waist circumference and blood pressure, collectively named metabolic syndrome (MetS) [4,5], has been linked to the development of sarcopenia in older adults. For instance, based on the cross-sectional NHANES database, lower muscle mass is associated with a higher risk of pre-diabetes and insulin sensitivity [6], and an inverse relationship between skeletal muscle mass and metabolic syndrome development has been reported in a 7-year longitudinal study in a large population of men and women [7].

Healthy dietary habits and engagement in regular physical activity have been advocated by major health organisations in order to mitigate the age-related loss of muscle mass [8,9]. In this respect, adherence to healthy dietary patterns, including the Mediterranean Diet and the Dietary Approach to Stop Hypertension (DASH), has been linked to beneficial health effects in older adults [10,11]. A recent investigation showed that adherence to healthy dietary patterns such as the Mediterranean diet can mitigate the age-related decline in muscle mass [12]. Importantly, a key feature of healthy diets is the high fibre content related to fruit, vegetable, legume and whole-grain intake [13,14,15]. Dietary fibre has important physiological effects on glucose and lipid metabolism, and several studies have documented a protective effect against diabetes and cardiovascular disorders. Unfortunately, the majority of the general population, including older adults, has a dietary fibre intake (FI) below recommended daily amounts [16,17].

Although dietary FI has an influence on markers of metabolic health and cardiovascular disorders [18,19,20,21], inconclusive findings have been reported regarding the effects on body composition [22,23,24,25]. Furthermore, there is a paucity of information about the potential impact of dietary fibres on the age-related loss of muscle mass and functional decline. Interestingly, it is suggested that dietary fibre has a beneficial effect on the systemic inflammatory milieu. Specifically, dietary fibres may delay glucose absorption and alter gut microflora, which may attenuate the release of pro-inflammatory cytokines [26,27]. Thus, given the detrimental effect of inflammation on muscle mass and protein synthesis [28,29], it can be suggested that an adequate intake of dietary fibre may promote the maintenance of muscle mass in older adults.

Importantly, in order to depict the true impact of FI on muscle mass, other features of a healthy diet must be considered. For example, we have previously shown that adherence to recommended intakes of proteins is associated with a higher muscle mass in older women [8]. Moreover, as regular exercise has established effects on the maintenance of muscle mass and function, information on objectively assessed physical activity (PA) levels needs to be considered when exploring the impact of diet. In this context, the NU-AGE project, comprising data on dietary intake and body composition from over a thousand European older men and women, offers a unique opportunity to close the current evidence gap on the significance of dietary fibre for the maintenance of muscle mass and function in older adults.

Therefore, the aim of the present study was to examine the links between FI, skeletal muscle mass and physical function in a large cohort of older men and women from the NU-AGE study.

## 2. Materials and Methods

### 2.1. Subjects

The present study included older men and women (*n* = 981; age 65–79 years) recruited within the frame of the NU-AGE project, a multi-centre study exploring determinants of healthy aging across five European countries. At screening, subjects fulfilling frailty criteria were excluded [30]. A detailed description of the recruitment process and study design has been described elsewhere [30,31]. In the context of the present study, data from France were not included due to a lack of information on accelerometer-based PA. Local ethical approval was provided by the Independent Ethics Committee of the Sant’Orsola-Malpighi Hospital Bologna (Italy-103/2011/U/Sper), the National Research Ethics Committee–East of England (United Kingdom-12/EE/0109), the Wageningen University Medical Ethics Committee (Netherlands-11/41 NU-AGE) and the Bioethics Committee of the Polish National Food and Nutrition Institute (Poland). Written informed consent was obtained from participants before starting and the study was conducted in accordance with the standards set by the Declaration of Helsinki. The trial was registered at clinicaltrials.gov (NCT01754012).

### 2.2. Assessment of Body Composition

Height and weight were measured using standardised procedures and body composition was assessed using whole body Dual energy X-ray absorptiometry (DXA) as described elsewhere [32]. DXA scans were performed by trained technicians according to state-of-the-art technique and manufacturer recommendations. The analytical program defined six corporeal regions. Total and regional fat and lean masses were derived. Skeletal muscle mass index (SMI, %) was derived as previously described [32,33,34]: (appendicular lean mass (kg)/total body weight (kg)) × 100.

### 2.3. Assessment of Dietary Intake

Dietary intake was assessed using a 7-day food record. Consumed foods were coded according to standardised procedures and translated into nutrients using software exploiting local food composition tables (NEVO 2011 in The Netherlands, WISP in the UK, INRAN and IEO in Italy, and NFNI in Poland). Macronutrient intakes and the amount of dietary FI were retrieved and averaged per day. In addition, adherence to healthy dietary patterns was obtained using the NU-AGE Food Based Dietary score (Mediterranean-type diet) [35]. Participants having a protein intake above 1.1 g/kg of body weight were classified as meeting the recommended amount of protein intake for older people [36].

### 2.4. Assessment of Physical Function

The short-physical performance battery (SPPB), including measures of gait speed, chair stand and balance, was conducted and test scores ranging from 0 (the worst performance) to 12 (the best performance) were derived as previously described [37]. Handgrip strength normalised by body weight was assessed using standardised procedures with a Jamar handheld dynamometer (Patterson Medical, Warrenville, IL, USA).

### 2.5. Assessment of Adherence to Physical Activity Guidelines Using Accelerometry

Physical activity was monitored using a waist-mounted Actigraph accelerometer (GT3x activity monitor, Actigraph, Pensacola, FL, USA) for a period of one week. In short, a minimum of 4 days with at least 10 h per day of wear time was required for inclusion and periods of non-wear time were determined as previously described [38]. Weekly time spent in moderate-to-vigorous PA (MVPA) was retrieved based on an established accelerometer count cut-point (≥2020 counts per minute) [39]. Participants spending a daily average of ≥22 min in MVPA, approximate to 150 min/week, were classified as adhering to the PA guidelines.

### 2.6. Assessment of Metabolic Risk

Waist circumference (WC) was measured to the nearest 0.1 cm. Systolic and diastolic blood pressures were assessed using an automated electronic blood pressure monitor as previously described [40]. All biochemical analyses including blood glucose (mmol/L), total cholesterol, HDL and LDL-cholesterol, triglycerides, adiponectin, high-sensitive C-reactive protein (hs-CRP) and white blood cell count (WB) were measured in one centre using standard methodologies. Participants were classified as having high and low metabolic risk based on the International Diabetes Federation definition of the metabolic syndrome [4].

### 2.7. Statistics

Data are presented as mean ± standard deviation, unless indicated otherwise. All variables were checked for normality and transformed when necessary. Differences in continuous variables between sex-specific SMI tertiles were assessed by one-way ANOVA, adjusted for multiple comparisons by Sidak-Holm correction. Corresponding differences in proportions were determined by chi-square tests. Differences between sex-specific groups of metabolic risk (with and without MetS) were assessed by independent samples *t*-test and chi-square tests. Given the well-established impact of gender on skeletal muscle mass, sex-specific general linear models were employed to determine the influence on SMI and physical function outcomes by groups of FI while adjusting for age, study centre, total energy intake and MetS (main effect and FI × MetS interaction) in model 1; and protein intake, adherence to healthy diet and PA guidelines in model 2. Effect sizes were estimated for each model (partial eta: η²p). Based on our sample size, an a priori power calculation revealed that small-to-moderate effect sizes are detectable with a power of > 80% with alpha set to 0.05. All analyses were conducted using SPSS version 26.

## 3. Results

A total of 981 European participants (mean age 71 ± 4 years, 58% female) were included in the final analysis. Subject characteristics including anthropometrics, metabolic and inflammatory biomarkers across sex-specific tertiles of SMI are presented in Table 1. The average SMI in men and women was 30.6 ± 3.1% and 24.4 ± 2.8%, respectively. About 54% of the total population fulfilled the guidelines of weekly MVPA time (men: 64% vs. women: 47%, *p* < 0.001) and 41% were classified with MetS.

Men and women belonging to the highest SMI tertile had a significantly lower prevalence of MetS and higher SPPB score, handgrip strength and adherence to PA guidelines compared to lower tertiles (Table 1). Additionally, belonging to the highest SMI tertile was related to lower waist circumference, as well as lower levels of blood glucose, triglycerides and hsCRP together with higher levels of HDL and adiponectin compared to those in lower tertiles (Table 1). Total fat, trunk, android and gynoid fat masses were significantly lower for those belonging to the highest SMI tertile compared to the other two tertiles (Table 1). Notably, participants with MetS had lower SMI, SPPB score (only female) and handgrip strength compared to those without MetS (Table 2).

The total energy intake and distribution of macronutrient intakes of the whole study sample were 1808 ± 425 kcal (men: 2019 ± 451 kcal vs. women: 1653 ± 327 kcal, *p* < 0.001), 49 ± 6 E% carbohydrates, 35 ± 6 E% fat and 17 ± 3 E% protein. The total fibre intake for the whole sample was 22.5 ± 7.8 g/day with median intakes of 23.5 (IQR 18.1–28.6) and 20.3 (IQR 16.3–24.8) for men and women, respectively. In women, 24.0% adhered to the recommended fibre intake (25 g/d), while the corresponding proportion in male was 20.2% (30 g/d). In both sexes, participants belonging to the third tertile of SMI had significantly higher fibre and protein intakes as well as a higher total energy intake compared to those in lower tertiles (Table 3).

**Figure 1:**
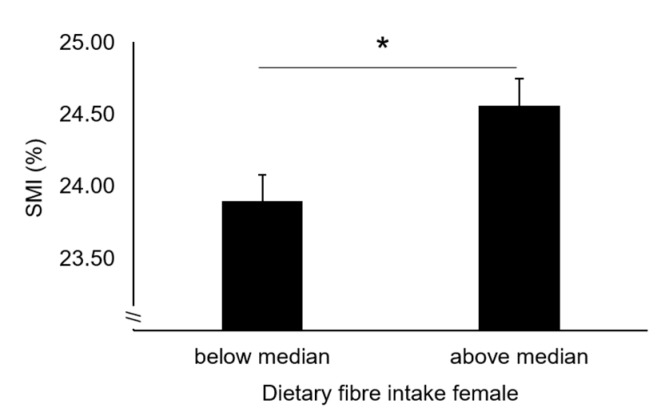
Skeletal muscle mass in older women below and above median FI. Data are estimated marginal means ± SEM adjusted for age, recruiting centre, total energy intake and prevalence of metabolic syndrome. SMI, Skeletal Muscle Mass Index. * *p* < 0.05.

Based on general linear models, older women with FI above the median value had significantly higher SMI (24.6 ± 0.2% vs. 23.9 ± 0.2%, *p* = 0.003, η^2^p = 0.016) compared to those under the median FI value (Figure 1), after adjustment for metabolic risk status (model 1).

The influence of FI on SMI remained after further adjustment for adherence to the PA guidelines, protein intake and adherence to a healthy diet (24.7 ± 0.2% vs. 24.2 ± 0.1%, *p* = 0.011, η^2^p = 0.012).

**Figure 2:**
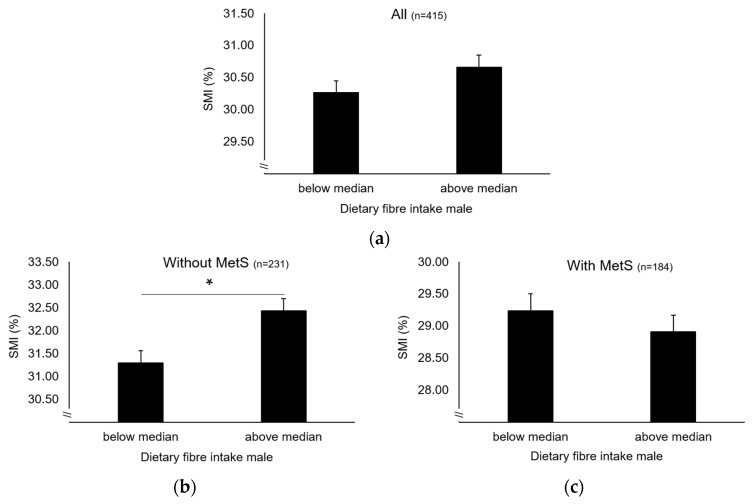
Skeletal muscle mass in older men below and above median FI (**a**) and stratified by MetS (**b** and **c**). Data are estimated marginal means ± SEM adjusted for age, recruiting centre and total energy intake (and prevalence of MetS in all). SMI, Skeletal Muscle Mass Index. * *p* < 0.05.

While no main effect was observed (Figure 2a), a significant interaction between MetS and FI on SMI was observed in older men (*p* = 0.009) (model 1). After stratification by MetS, a significant main effect of FI on SMI was observed in older men without MetS (32.4 ± 0.3% vs. 31.3 ± 0.3%, *p* = 0.005, η^2^p = 0.035) (Figure 2b), which remained after further covariate adjustments. In contrast, no corresponding effect was shown in those with MetS (Figure 2c). Interestingly, a significant difference in FI was observed between older men without and with MetS (25.8 ± 9.8 g vs. 22.5 ± 7.0 g, *p* < 0.001) but not in older women (21.5 ± 6.5 g vs 20.8 ± 6.7 g, NS). Given the proposed association between FI and the inflammatory status, we examined hs-CRP levels between groups of FI. The data show that participants with FI above median had significantly lower hs-CRP level compared to those below (1.32 ± 1.96 mg/L vs. 1.82 ± 2.30 mg/L in men and 1.27 ± 1.81 mg/L vs. 2.06 ± 2.58 mg/L in women, *p* < 0.05).

There was no main effect of FI on the physical function parameters, including SPPB score and handgrip strength and, no interactions between FI and MetS were observed.

## 4. Discussion

A main finding from the study was the beneficial impact of FI on skeletal muscle mass in a cohort of older European men and women, which is independent of adherence to a healthy dietary pattern, and guidelines for protein intake and objectively assessed physical activity level.

While a large body of research has focused on the role of dietary protein intake in the regulation of muscle mass, less attention has been given to the impact of healthy diets in general, particularly FI. Our findings indicate that a low FI is detrimental for the maintenance of muscle mass in older adults. This is in line with the PREDIMED-plus study reporting lower FI in participants with low muscle mass compared to those with higher muscle mass [12]. Dietary fibre’s impact on muscle mass may be explained by its action on gut microflora and circulating free fatty acid concentration, which may attenuate systemic inflammation [41,42]. Indeed, our data show that compared to those with lower FI (below median), participants with high FI had lower levels of CRP, an established clinical marker of systemic inflammation. Given the proposed interaction between systemic inflammation and the regulation of muscle mass, we suggest that a link between FI and muscle mass can be mediated through dietary fibre-induced alterations in the systemic environment.

The present study addressed the potential interaction between FI and metabolic risk on muscle mass. While such an interaction was evident in older men, the metabolic risk status did not influence the link between FI and muscle mass in older women. Importantly, this sex-specific interaction may be partly explained by the fact that older men with MetS have a significantly lower FI compared to men without MetS. In contrast, FI did not differ between older women with and without MetS. Given this, it may be hypothesized that the FI observed in older men with MetS is insufficient to offset the detrimental effects of metabolic disturbances on regulation of muscle mass.

Altogether, our findings support the promotion of increased FI in older adults for the maintenance of muscle mass. We and others have previously highlighted the beneficial impact of adequate protein intake on muscle mass in older adults [8,43,44]. In this respect, the fact that the impact of protein intake on muscle mass was accounted for in the current study further strengthens the proposed beneficial role of adequate FI in the regulation of muscle mass.

Physical activity is another important lifestyle factor with the potential to readily influence skeletal muscle mass in older adults. In the present study, the association between FI and muscle mass was evident regardless of the objectively assessed adherence to PA guidelines, which highlights the importance of adequate FI as an independent lifestyle factor in the promotion of healthy aging.

Of note, participants belonging to the highest SMI tertile had lower levels of CRP and higher levels of the anti-inflammatory biomarker adiponectin, highlighting interplay between systemic inflammatory milieu and muscle mass regulation. In addition, differences in blood lipid profile were observed across SMI tertiles, with those in the highest tertile showing a more favorable profile (higher HDL-cholesterol and lower triglyceride levels) compared to the rest of participants. Further research is needed to elucidate mechanistic links between blood lipid profile, inflammatory status, and dietary habits in older adults.

In contrast to the link observed between FI and muscle mass, there was no relationship between FI and physical function parameters. These contrasting findings suggest that physical function is influenced by other factors than muscle mass alone. For example, age-related changes in strength capacity have been shown to be weakly related to changes in muscle mass [45]. However, the FI-related link to muscle mass may benefit long-term maintenance of muscle function and delay loss of independence.

The main findings of the present study are strengthened by (1) the use of a large cohort of older adults covering diverse ethnic and cultural differences, which allows for generalisation to broader European populations; and (2) state-of-the-art assessment of muscle mass using DXA, together with the use of dietary record and objective measurement of PA. The following limitations should be considered: given its cross-sectional design, making inference on causality is not possible and residual confounding from unmeasured variables cannot be ruled out. Further, dietary fibre is a diverse category of insoluble and soluble components which have different physiological effects [46]. Potential variations in analysis of food items between countries are likely to produce slight variations in assessment of absolute fibre intakes. However, given that the present study examines links between higher/lower fibre intakes and muscle mass, rather than comparing absolute intakes of fibre between countries, such variations in FI are unlikely to alter the study conclusions.

## 5. Conclusions

In conclusion, the NU-AGE study highlights the importance of an adequate intake of dietary fibre within dietary strategies to combat the age-related decline in muscle mass and promote healthy aging.

## Figures and Tables

**Table 1:** Characteristics of the study sample according to sex-specific tertiles of skeletal muscle mass index (SMI).

	SMI Male			SMI Female		
	Tertile 1	Tertile 2	Tertile 3	Tertile 1	Tertile 2	Tertile 3
n	139	138	138	189	188	189
SMI, %	≤29.1	>29.1–≤31.8	>31.8	≤ 23.2	>23.2–≤25.6	> 25.6
**Basic Characteristics**						
Age, y	72 ± 4	71 ± 4	70 ± 4 *	71 ± 4	71 ± 4	71 ± 4
Weight, kg	89.3 ± 11.6	81.6 ± 10.4 *	75.8 ± 9.1 *^#^	76.1 ± 11.4	68.0 ± 10.0 *	63.0 ± 8.2 *^#^
Height, cm	172 ± 7	173 ± 6	174 ± 6	159 ± 7	159 ± 6	162 ± 7 *^#^
Years full education, y	13 ± 4	13 ± 4	13 ± 3	12 ± 4	12 ± 3	12 ± 3
Smoking, % never	30.2	39.1	44.2	60.3	62.2	61.9
Medication, % yes	89.2	81.9	60.9	83.1	80.9	68.8
PA. guidelines, % yes	56.1	60.9	74.6	39.7	48.9	51.9
**Physical function**						
Handgrip, kg/kg BW	0.44 ± 0.08	0.49 ± 0.09 *	0.56 ± 0.09 *^#^	0.32 ± 0.08	0.38 ± 0.08 *	0.43 ± 0.10 *^#^
SPPB, score	11.5 ± 0.9	11.4 ± 1.0	11.8 ± 0.8 *^#^	10.8 ± 1.5	11.3 ± 1.3 *	11.5 ± 0.9 *
**Metabolic Risk Factors**						
WC, cm	105.5 ± 9.1	97.8 ± 8.6 *	90.2 ± 7.4 *^#^	94.9 ± 9.9	88.1 ± 9.7 *	81.6 ± 8.1 *^#^
BMI, kg/m^2^	29.7 ± 3.3	27.0 ± 2.8 *	24.8 ± 2.6 *^#^	29.8 ± 3.8	26.5 ± 3.5 *	23.9 ± 3.0 *^#^
DBP, mmHg	76 ± 10	76 ± 11	78 ± 10	75 ± 10	74 ± 11	73 ± 11
SBP, mmHg	142 ± 17	140 ± 19	141 ± 18	141 ± 21	140 ± 22	138 ± 21
MetS, % yes	73.4	44.2	15.2	55.6	42.0	20.1
Glucose, mmol/L	6.14 ± 1.02	5.69 ± 0.74 *	5.37 ± 0.76 *^#^	5.67 ± 0.82	5.39 ± 0.64 *	5.24 ± 0.57 *
Triglycerides, mmol/L	1.21 ± 0.55	1.05 ± 0.45 *	0.97 ± 0.43 *	1.15 ± 0.46	1.07 ± 0.46	0.96 ± 0.40 *
TC, mmol/L	1.87 ± 0.40	1.92 ± 0.37	2.02 ± 0.40 *	2.16 ± 0.40	2.18 ± 0.42	2.28 ± 0.41 *
HDL-cholesterol, g/L	0.46 ± 0.12	0.49 ± 0.13	0.57 ± 0.15 *^#^	0.62 ± 0.16	0.64 ± 0.17	0.72 ± 0.19 *^#^
LDL-cholesterol, g/L	1.17 ± 0.37	1.22 ± 0.33	1.25 ± 0.36	1.32 ± 0.37	1.33 ± 0.39	1.37 ± 0.38
Adiponectin, µg/mL	7.96 ± 4.47	9.63 ± 6.23	9.87 ± 6.63 *	14.93 ± 8.44	15.68 ± 9.24	18.01 ± 9.81 *
hs-CRP, mg/L	2.0 ± 2.4	1.7 ± 2.3	1.1 ± 1.6 *^#^	2.2 ± 2.7	1.5 ± 1.9 *	1.3 ± 2.0 *^#^
WBC, % total count	5.9 ± 1.2	5.7 ± 1.5	5.7 ± 1.4	5.8 ± 1.4	5.8 ± 1.5	5.9 ± 1.5
**Fat mass**						
Total fat mass, kg	30.4 ± 5.8	22.8 ± 4.9 *	15.4 ± 4.6 *^#^	34.2 ± 7.2	26.2 ± 5.5 *	20.3 ± 5.0 *^#^
Trunk fat mass, kg	19.3 ± 3.9	13.9 ± 3.3 *	8.8 ± 3.1 *^#^	18.1 ± 4.2	13.5 ± 3.3 *	9.9 ± 3.0 *^#^
Android fat mass, kg	3.7 ± 0.8	2.5 ± 0.7 *	1.6 ± 0.6 *^#^	3.3 ± 0.9	2.3 ± 0.6 *	1.7 ± 0.5 *^#^
Gynoid fat mass, kg	4.1 ± 0.8	3.2 ± 0.7 *	2.4 ± 0.7 *^#^	5.8 ± 1.3	4.4 ± 0.9 *	3.8 ± 0.8 *^#^

Continuous data are expressed as mean ± SD. SMI, skeletal muscle mass index. BW, body weight. PA, physical activity. SPPB, short physical performance battery. WC, waist circumference. DBP, diastolic blood pressure. SBP, systolic blood pressure. MetS, metabolic syndrome. TC, total cholesterol. HDL, high-density lipoprotein. LDL, low-density lipoprotein. hs-CRP, high-sensitivity c-reactive protein. WBC, white blood cell count. * *p* < 0.05 vs. T1. ^#^ *p* < 0.05 vs. T2.

**Table 2:** Skeletal muscle mass index and physical function in participants with and without MetS.

	MetS Male		MetS Female	
	No	Yes	No	Yes
n (%)	231 (56%)	184 (44%)	344 (61%)	222 (39%)
SPPB, score	11.6 ± 0.9	11.5 ± 0.9	11.3 ± 1.3	11.0 ± 1.3 *
Handgrip, kg/kg BW	0.52 ± 0.10	0.47 ± 0.08 *	0.40 ± 0.10	0.35 ± 0.09 *
SMI, %	32.0 ± 2.9	28.9 ± 2.4 *	25.2 ± 2.8	23.2 ± 2.4 *

Data are expressed as mean ± SD. MetS, metabolic syndrome. SPPB, short physical performance battery. BW, body weight. SMI, skeletal muscle mass index. * *p* < 0.05 between sex-specific groups.

**Table 3:** Fibre, protein and total energy intake and adherence to healthy diet in sex-specific tertiles of skeletal muscle mass index.

	SMI Male			SMI Female		
	T1	T2	T3	T1	T2	T3
Total energy intake, kcal	1902 ± 418	1968 ± 413	2189 ± 473 *^#^	1571 ± 314	1657 ± 304 *	1730 ± 345 *^#^
Fibre, g/d	21.8 ± 7.7	23.6 ± 8.7	27.5 ± 9.2 *^#^	19.6 ± 5.8	20.6 ± 6.3	23.4 ± 7.0 *^#^
Protein, g/kg BW	0.90 ± 0.23	0.99 ± 0.23 *	1.15 ± 0.25 *^#^	0.91 ± 0.24	1.02 ± 0.21 *	1.12 ± 0.24 *^#^
Adherence healthy diet, %	48.9 ± 9.1	49.4 ± 8.4	48.9 ± 9.3	50.2 ± 9.7	50.9 ± 9.3	50.9 ± 9.4

Data expressed as mean ± SD. SMI, skeletal muscle mass index. BW, body weight. * *p* < 0.05 vs. T1. ^#^ *p* < 0.05 vs. T2.
